# Supplementary figures and images for: High-throughput analysis of lung immune cells in a combined murine model of agriculture dust-triggered airway inflammation with rheumatoid arthritis
Source: PLoS One. 2021 Feb 12;16(2):e0240707. doi: 10.1371/journal.pone.0240707 (PMC7880471; doi:10.1371/journal.pone.0240707)

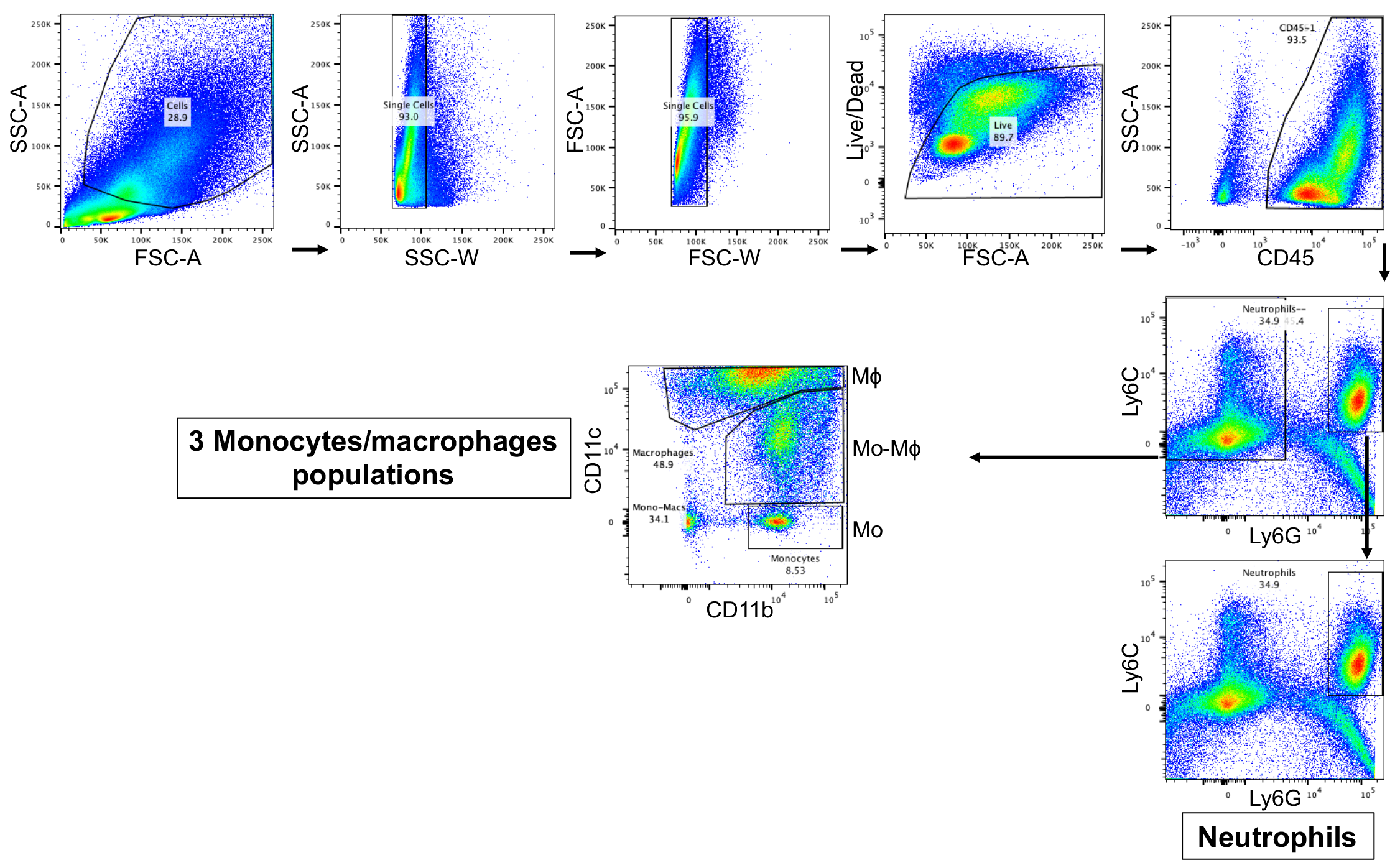

Supplement: S1 Fig — Neutrophils were sorted from lung digests as live, singlets, CD45+, non-lymphocytes, Ly6C+ and Ly6G+. Three monocyte/macrophage populations were sorted from lung digests as live, singlets, CD45+, non-lymphocytes, Ly6C–and Ly6G–, and identified as separate populations with variable expression of CD11b and CD11c as: (1) macrophages (CD11chigh, CD11bvariable), (2) monocytes-macrophages (CD11cintermediate,CD11bhigh), and (3) monocytes (CD11c–, CD11bhigh). (TIF) [file pone.0240707.s001.tif]

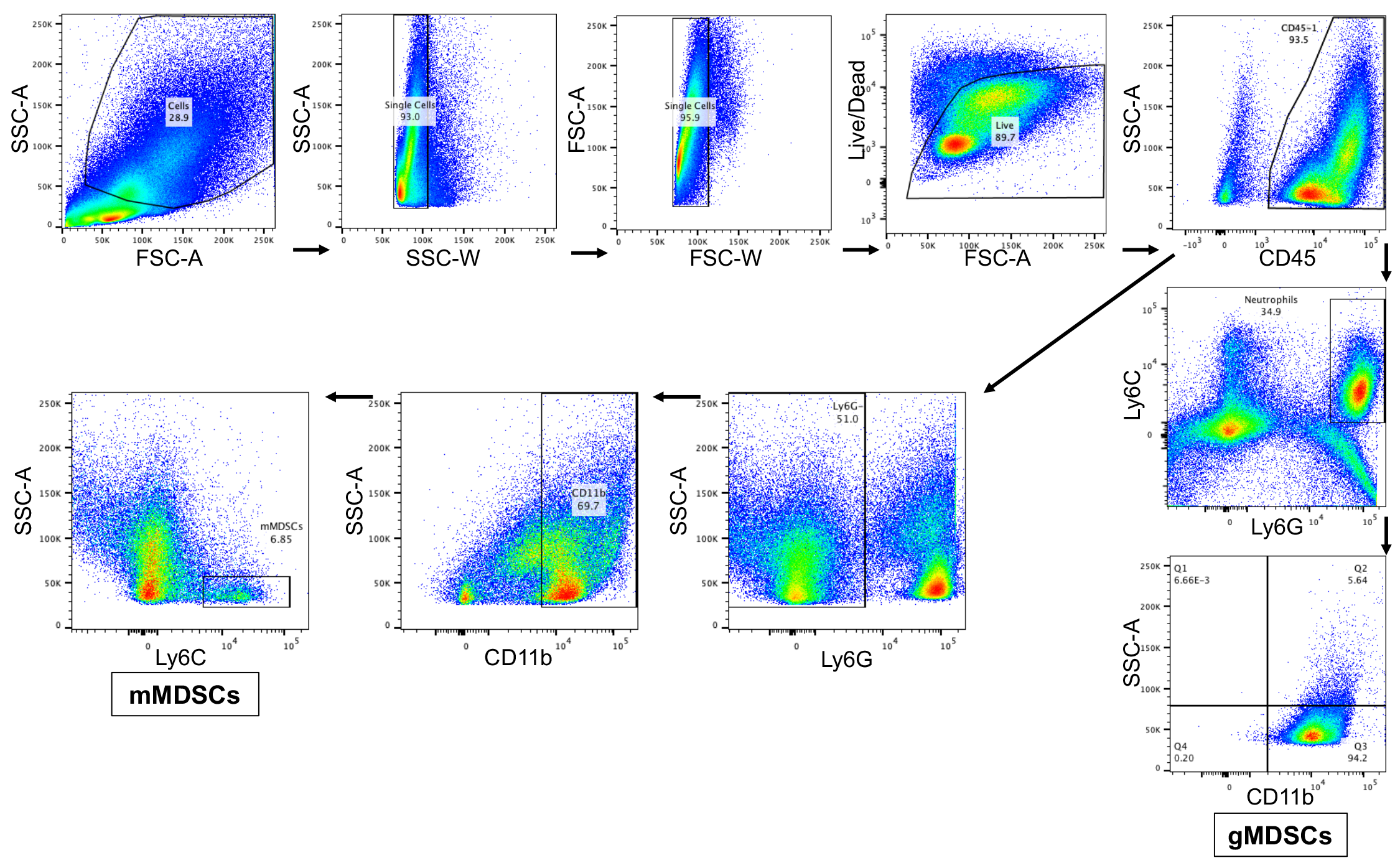

Supplement: S2 Fig — Granulocytic (g) MDSCs were identified as live, singlets, CD45+, non-lymphocytes that were Ly6C+ Ly6G+ CD11b+ SSChigh. Whereas monocytic (m) MDSCs were identified as live, singlets, CD45+, non-lymphocytes that were Ly6G– CD11b+ Ly6C+ SSClow. (TIF) [file pone.0240707.s002.tif]
