# Supplementary material for: High-throughput analysis of lung immune cells in a combined murine model of agriculture dust-triggered airway inflammation with rheumatoid arthritis
Source: PLoS One. 2021 Feb 12;16(2):e0240707. doi: 10.1371/journal.pone.0240707 (PMC7880471; doi:10.1371/journal.pone.0240707)
Supplement: S1 Table — (PDF) [file pone.0240707.s003.pdf]

S1 Table. The top 10 genes uniquely identified to neutrophil subtypes with mean UMI count, log2 fold-change and adjusted p value as compared to all other $CD45^{+}$ lung cell clusters.

Cluster 8 – Inflammatory Neutrophils

Gene	Mean UMI Count	Log2 fold change	Adjusted p value
Ccl3	124.78	6.35	1.33E-61
Ccl4	223.60	6.06	3.09E-54
Il23a	1.69	6.06	1.15E-32
Gm39459	1.07	6.03	2.06E-37
Upp1	3.30	5.54	1.77E-38
Marcksl1	17.46	4.98	6.02E-30
Il1rn	24.09	4.88	1.41E-27
Csf1	1.45	4.82	7.22E-23
Ier3	56.06	4.82	2.37E-27
Ccrl2	18.97	4.81	1.98E-26

Cluster 3 – Granulocyte Myeloid-Derived Suppressor Cells/Autoreactive Neutrophils)

Gene	Mean UMI Count	Log2 fold change	Adjusted p value
Ly6g	1.99	6.54	1.32E-80
Retnlg	67.42	6.14	6.00E-85
Ifit3b	1.78	6.03	5.45E-69
Ifit3	7.17	5.82	9.24E-72
S100a8	488.82	5.54	1.26E-67
Mrgpra2b	1.31	5.37	5.80E-52
S100a9	465.22	5.31	4.23E-61
Lrg1	9.86	5.22	3.94E-56
Stfa2l1	2.58	5.17	2.27E-44
Mmp8	3.25	5.16	1.60E-51

Cluster 4 – Resident/Transitional Neutrophils

Gene	Mean UMI Count	Log2 fold change	Adjusted p value
Csf3r	6.12	4.24	1.26E-20
Il1r2	4.21	4.20	1.32E-19
Slc40a1	1.25	4.17	6.12E-18
H2-Q10	2.90	4.07	7.74E-18
4833407H14Rik	1.11	4.01	4.51E-16
Gm34084	1.59	3.99	2.27E-16
Cxcr2	3.64	3.91	2.31E-16
Lmnb1	7.54	3.91	1.73E-16
F630028O10Rik	1.47	3.88	7.75E-15
Fgl2	3.58	3.87	9.33E-16
