# Supplementary material for: High-throughput analysis of lung immune cells in a combined murine model of agriculture dust-triggered airway inflammation with rheumatoid arthritis
Source: PLoS One. 2021 Feb 12;16(2):e0240707. doi: 10.1371/journal.pone.0240707 (PMC7880471; doi:10.1371/journal.pone.0240707)
Supplement: S2 Table — (PDF) [file pone.0240707.s004.pdf]

S2 Table. The top 10 genes uniquely identified to monocyte, macrophage and DC subtypes with average UMI count, log2 fold-change and adjusted p value compared to all other CD45$^+$ lung cell clusters.

Cluster 1 – Airspace Macrophages

**Gene**	**Mean UMI Count**	**Log2 fold change**	**Adjusted p value**
Ear1	4.10	1.98	8.33E-17
Ear2	10.07	1.94	2.80E-16
Ltc4s	1.64	1.85	8.38E-15
S100a1	2.35	1.72	8.44E-13
Il18	1.65	1.69	2.06E-12
Fabp1	3.94	1.65	2.08E-11
Slpi	5.88	1.62	1.68E-11
Krt19	1.26	1.55	2.50E-10
Lyz2	125.33	1.52	3.81E-10
Cd63	2.72	1.45	4.02E-09

Cluster 2 – Differentiating Recruited Macrophages

**Gene**	**Mean UMI Count**	**Log2 fold change**	**Adjusted p value**
Net1	1.08	1.54	2.37E-07
Tcf7l2	2.40	1.40	3.83E-06
Abcg1	6.06	1.33	1.50E-05
Serpine1	1.12	1.34	1.87E-05
Pla2g15	1.08	1.31	2.55E-05
Tgm2	2.24	1.29	3.51E-05
Ptpn12	3.05	1.28	4.24E-05
Mrc1	4.14	1.27	5.25E-05
Glul	1.67	1.26	5.94E-05
Ncoa4	1.81	1.25	6.72E-05

Cluster 5 – Recruited Macrophages

**Gene**	**Mean UMI Count**	**Log2 fold change**	**Adjusted p value**
Inhba	2.78	1.96	9.83E-10
Cxcl3	5.01	1.86	2.81E-08
Hmox1	1.93	1.56	8.97E-06
Tgm2	2.65	1.53	6.20E-06
Rpn1	1.29	1.53	7.59E-06
Car4	3.64	1.50	1.35E-05
Mmp19	1.42	1.50	1.50E-05
Anxa4	2.38	1.39	7.51E-05
Mt2	2.31	1.37	1.86E-04
Pdia6	1.94	1.35	1.40E-04

Cluster 11 – Resident Pro-Resolving Interstitial Macrophages

**Gene**	**Mean UMI Count**	**Log2 fold change**	**Adjusted p value**
Apoe	11.75	3.91	4.99E-20
Ccl2	1.39	3.96	3.64E-18
Fcgr2b	1.97	3.36	2.36E-17

C1qc	1.09	3.93	4.52E-15
Cxcl16	1.78	3.00	2.07E-12
C1qb	1.77	3.56	2.43E-12
C1qa	1.35	3.54	2.02E-10
Il6	1.03	2.82	2.70E-09
Cxcl1	3.68	2.76	1.93E-08
Saa3	1.29	3.13	1.55E-05

Cluster 14 – Proliferative Airspace Macrophages

**Gene**	**Mean UMI Count**	**Log2 fold change**	**Adjusted p value**
Nusap1	1.16	8.12	2.30E-93
Top2a	2.81	7.05	5.15E-82
Birc5	1.50	6.95	1.33E-78
Rrm2	1.12	7.00	3.04E-66
Pclaf	1.80	6.48	5.23E-63
Ube2c	1.56	6.50	2.80E-60
Cenpf	1.04	6.33	9.43E-52
Mki67	1.72	5.79	2.31E-51
Stmn1	2.98	4.95	7.51E-39
Cenpa	1.06	3.97	6.76E-22

Cluster 12 – Inflammatory Monocytes

**Gene**	**Mean UMI Count**	**Log2 fold change**	**Adjusted p value**
F13a1	3.09	6.53	2.53E-88
Ms4a4c	2.26	5.51	4.15E-65
Ly6c2	14.74	4.83	1.69E-49
Aif1	1.03	4.99	3.75E-46
Vcan	1.01	4.89	1.13E-45
Plac8	19.22	4.63	8.02E-45
Ccr2	3.55	4.65	1.36E-44
Ms4a6c	3.98	4.27	3.68E-36
Ifitm3	17.05	3.90	5.68E-30
S100a4	9.87	3.90	1.41E-28

Cluster 10 – Dendritic Cells

**Gene**	**Mean UMI Count**	**Log2 fold change**	**Adjusted p value**
Siglech	1.04	6.48	6.29E-30
Hes1	1.37	4.14	1.37E-14
Tcf4	1.30	3.54	2.39E-12
Rnase6	1.03	3.34	1.05E-10
Pou2f2	1.42	3.22	9.56E-10
Plac8	12.12	3.04	2.80E-08
Id3	1.67	3.03	7.51E-08
Bst2	2.53	2.61	1.46E-05
Ifitm6	1.16	2.57	1.96E-05
Ly6a	1.43	2.54	4.03E-05
