# Supplementary material for: High-throughput analysis of lung immune cells in a combined murine model of agriculture dust-triggered airway inflammation with rheumatoid arthritis
Source: PLoS One. 2021 Feb 12;16(2):e0240707. doi: 10.1371/journal.pone.0240707 (PMC7880471; doi:10.1371/journal.pone.0240707)
Supplement: S3 Table — (PDF) [file pone.0240707.s005.pdf]

S3 Table. The top 10 genes uniquely identified to lymphocytes subtypes with average UMI count, log2 fold-change and adjusted p value compared to all other CD45^{+} lung cell clusters.

Cluster 6 – T Lymphocytes

Gene	Mean UMI Count	Log2 fold change	Adjusted p value
Lef1	1.65	7.04	2.56E-104
Igfbp4	1.92	5.75	1.40E-65
Dusp10	1.39	5.62	2.40E-65
Tcf7	1.37	5.61	1.46E-64
Cd3d	2.49	4.78	1.69E-48
Cd3e	1.22	4.87	1.50E-46
Trac	1.39	4.76	9.92E-44
Trbc2	4.67	4.62	2.66E-42
S1pr1	1.06	4.49	2.22E-38
Gm8369	1.33	4.44	4.35E-38

Cluster 13 – CD4+ T Lymphocytes

Gene	Mean UMI Count	Log2 fold change	Adjusted p value
Icos	2.22	5.87	2.37E-35
Thy1	2.44	5.04	1.13E-24
Cd3g	3.33	4.81	3.06E-22
Ikzf2	1.65	5.28	1.26E-19
Trac	2.08	4.60	4.94E-19
Maf	1.08	4.87	1.31E-18
Ctla2a	2.11	4.61	1.62E-16
Cd3e	1.57	4.36	2.86E-16
Cd28	1.48	4.33	1.77E-15
Lat	2.11	4.10	3.79E-14

Cluster 7 – B Lymphocytes

Gene	Mean UMI Count	Log2 fold change	Adjusted p value
Ebf1	6.40	7.77	9.62E-136
Cd79a	6.62	7.63	3.23E-132
Ms4a1	2.87	7.32	1.25E-109
Cd79b	3.62	7.18	1.76E-109
Gm31243	1.31	7.64	9.25E-108
Ighd	2.33	7.29	9.25E-108
Fcmr	1.24	7.65	1.52E-105
Bank1	1.53	7.04	2.37E-98
Iglc2	4.59	6.90	4.05E-98
Ly6d	7.24	6.47	4.65E-90

Cluster 9 – NK Cells

Gene	Mean UMI Count	Log2 fold change	Adjusted p value
Ncr1	3.11	7.87	5.88E-100
Ccl5	99.32	7.51	5.92E-100
Gzma	30.93	7.70	1.13E-99

Nkg7	11.27	7.26	8.15E-95
Prf1	2.79	7.83	4.33E-90
Klre1	1.70	7.75	9.81E-90
Cma1	1.56	8.73	1.20E-86
Sytl3	1.38	7.25	5.58E-78
Gzmb	3.10	7.43	2.84E-74
Klrd1	3.73	6.45	4.13E-69
